# Micronized Palmitoylethanolamide Ameliorates Methionine- and Choline-Deficient Diet–Induced Nonalcoholic Steatohepatitis *via* Inhibiting Inflammation and Restoring Autophagy

**DOI:** 10.3389/fphar.2021.744483

**Published:** 2021-10-12

**Authors:** Jiaji Hu, Hanglu Ying, Jie Yao, Longhe Yang, Wenhui Jin, Huabin Ma, Long Li, Yufen Zhao

**Affiliations:** ^1^ Institute of Drug Discovery Technology, Ningbo University, Ningbo, China; ^2^ Technology Innovation Center for Exploitation of Marine Biological Resources, Third Institute of Oceanography, Ministry of Natural Resources, Xiamen, China

**Keywords:** nonalcoholic steatohepatitis, palmitoylethanolamide, oxidative stress, inflammation, autophagy

## Abstract

Nonalcoholic steatohepatitis (NASH) has become one of the serious causes of chronic liver diseases, characterized by hepatic steatosis, hepatocellular injury, inflammation and fibrosis, and lack of efficient therapeutic agents. Palmitoylethanolamide (PEA) is an endogenous bioactive lipid with various pharmacological activities, including anti-inflammatory, analgesic, and neuroprotective effects. However, the effect of PEA on nonalcoholic steatohepatitis is still unknown. Our study aims to explore the potential protective role of PEA on NASH and to reveal the underlying mechanism. In this study, the C57BL/6 mice were used to establish the NASH model through methionine- and choline-deficient (MCD) diet feeding. Here, we found that PEA treatment significantly improved liver function, alleviated hepatic pathological changes, and attenuated the lipid accumulation and hepatic fibrosis in NASH mice induced by MCD diet feeding. Mechanistically, the anti-steatosis effect of PEA may be due to the suppressed expression of ACC1 and CD36, elevated expression of PPAR-*α*, and the phosphorylation levels of AMPK. In addition, hepatic oxidative stress was greatly inhibited in MCD-fed mice treated with PEA via enhancing the expression and activities of antioxidant enzymes, including GSH-px and SOD. Moreover, PEA exerted a clear anti-inflammatory effect though ameliorating the expression of inflammatory mediators and suppressing the NLRP3 inflammasome pathway activation. Furthermore, the impaired autophagy in MCD-induced mice was reactivated with PEA treatment. Taken together, our research suggested that PEA protects against NASH through the inhibition of inflammation and restoration of autophagy. Thus, PEA may represent an efficient therapeutic agent to treat NASH.

## Introduction

Nonalcoholic steatohepatitis (NASH) is a severe form of nonalcoholic fatty liver disease (NAFLD), which has been one of the severest causes of liver disorder all over the world. NASH encompasses a range of symptoms, including hepatic steatosis, inflammation, ballooning, and fibrosis, with a great risk of progression to hepatocellular carcinoma (HCC). How NASH is initiated and progressed is largely unknown, as multiple factors are engaged in the pathogenesis of NASH. Currently, there is still a lack of specific and effective therapeutic strategies approved for the treatment of NASH. Thus, a promising medication to treat NASH is urgently needed (Bence and Birnbaum, 2020; Loomba et al., 2021).

Inflammatory response has been identified as one of the key mediators in the progress of NASH (Schuster et al., 2018). Kupffer cells, the resident macrophages in the liver, are usually activated to the pro-inflammatory condition upon the stimulation of pathogen-associated molecular patterns or damage-associated molecular patterns during the development of NASH (Huang et al., 2010). Activated Kupffer cells could secrete numerous pro-inflammatory cytokines and chemokines and recruit an increasing number of circulating monocytes into the injured liver, resulting in hepatic inflammation and aggravated steatohepatitis (Zhang et al., 2019). NLR family pyrin domain containing 3 (NLRP3) inflammasome is an intracellular multi-protein complex, which has been proved to have a key role in promoting inflammatory responses through secreting IL-1β and IL-18 (Swanson et al., 2019). As the activation of NLRP3 inflammasome is closely related to the progression of NASH, the strategy to suppress NLRP3 activation has been an efficient option to prevent NASH development (Mridha et al., 2017; Huang et al., 2021).

Autophagy is an evolutionarily conserved physiological process involved in eliminating damaged organelles and proteins to maintain intracellular homeostasis (Jiang and Mizushima, 2014). As an adaptive process in the preservation of liver health, autophagy plays an important role in the progression of all kinds of liver diseases (Madrigal-Matute and Cuervo, 2016). Abundant previous clinical and rodent studies have shown that autophagy was disordered in NASH; however, reactivating it could facilitate the clearance of lipids in hepatocytes to alleviate hepatic steatosis and hepatocellular injury during the development of NASH (Gonzalez-Rodriguez et al., 2014; Jin X. et al., 2020).

Palmitoylethanolamide (PEA) is an endogenous fatty acid amide as the ligand of multiple molecular targets, especially peroxisome proliferator-activated receptor-α (PPAR-α) (Lo Verme et al., 2005). Numerous studies have revealed that PEA has various pharmacological applications, including anti-inflammatory, analgesic, and neuroprotective effects (Skaper et al., 2015; Petrosino et al., 2016). However, the role of PEA in MCD-induced NASH and the underlying mechanisms have not been revealed. For this purpose, the present study was designed to investigate the effects of PEA in the treatment of NASH. PEA is available in two different formulations: micronized (m-PEA) and ultra-micronized (um-PEA) (Petrosino and Di Marzo, 2017). The m-PEA we used in this study has much better bioavailability and gastroenteric absorption than the normal PEA (Impellizzeri et al., 2019). We demonstrate that m-PEA administration protects against NASH by inhibiting the inflammatory response and restoring autophagy. These observations provide new insights into the interruption of the development of NASH.

## Materials and Methods

### Animal Experiments

**FIGURE 1:**
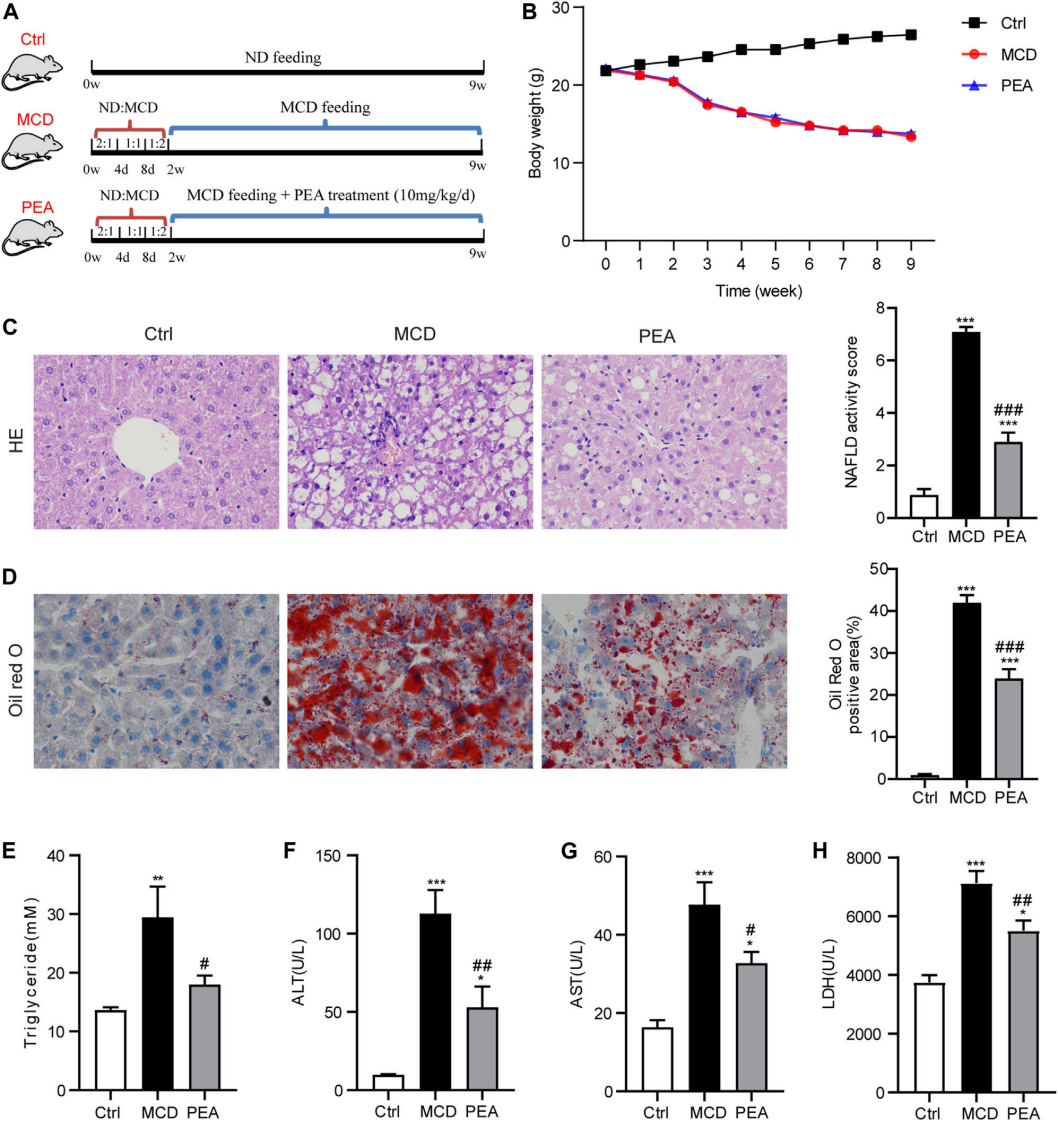
Effect of PEA on MCD-induced steatohepatitis in mice. **(A)** Experiment scheme for testing the preventive effects of PEA in the MCD-treated mice. **(B)** Body weight during the animal experiment. **(C)** Liver histological changes were determined by hematoxylin-eosin (HE) staining (× 400), and NAFLD activity score was quantified. **(D)** Hepatic lipid accumulation and distribution detected by Oil Red O staining (× 400), and Oil Red O-stained area was analyzed. **(E)** TG levels in the liver. **(F)** ALT levels in plasma. **(G)** AST levels in plasma. **(H)** LDH levels in plasma. Ctrl: the mice treated with standard diet; MCD: the mice treated with MCD diet; PEA: the mice treated with MCD diet and PEA. Values were expressed as the mean ± SEM, *n* = 6–8 for each group. *p < 0.05, **p < 0.01, ***p < 0.001 versus the Ctrl group; #*p* < 0.05, ##*p* < 0.01, ###*p* < 0.001 versus the MCD group.

Six-week-old adult male C57BL/6 mice were obtained from Vital River Laboratory Animal Technology Co., Ltd. (Beijing, China). Animal experiments were carried out in accordance with the approved guidelines involved in animal care by the Committee for Animal Research at Ningbo University. All mice were kept in a controlled temperature (21–23°C) and a constant humidity (55–60%) under a 12/12 h light/dark cycle, while food and water were provided ad libitum. The NASH model was established via feeding a methionine- and choline-deficient (MCD) diet, which can mimic the clinical characteristics of human NASH (Machado et al., 2015). The methionine and choline supplemented diet (normal diet, ND) and MCD diet (Trophic Animal Feed High-Tech Co., Ltd., Nantong, China) used in this study both contained 4.2 kJ/g energy, and the composition of the diet is shown in Supplementary Table S1. The energy in a normal diet was derived from 18% of protein, 60% of carbohydrate, and 22% of fat, while the MCD diet contained energy from 17% of protein, 61% of carbohydrate, and 22% of fat. To examine the preventive effect of PEA on MCD-induced NASH in mice, the mice were all randomly grouped as follows (*n* = 6–8/group): 1) Ctrl: control group fed with normal diet; 2) MCD: NASH model group fed with MCD diet and treated with the vehicle of PEA; 3) PEA: PEA group fed with MCD diet and intraperitoneal injection with PEA (10 mg/kg/day). The mice in the MCD group and PEA group were adaptively fed with a mixed diet of a normal diet and MCD diet for 2 weeks (2:1 for 4 days; 1:1 for 4 days; 1:2 for 6 days) and then received MCD feeding or MCD feeding + PEA treatment for 7 weeks. The experimental animal scheme is shown in Figure 1A.

### Reagents and Antibodies

The m-PEA was obtained from Wuxi Cima Science (Wuxi, China). Hematoxylin-Eosin (HE) staining kit was purchased from Solarbio Life Sciences (Beijing, China). Oil Red O was from Sigma-Aldrich (Shanghai, China). Tween-80 and PEG-400 were purchased from Sangon Biotech (Shanghai, China). Anti-PPAR-α antibody (#ab24509) and anti-CD68 antibody (#ab125212) were obtained from Abcam (Shanghai, China). Anti-caspase-1 antibody (clone 14F468) was purchased from Santa Cruz (Shanghai, China). Anti-GAPDH antibody (clone 1E6D9) was purchased from Proteintech (Wuhan, China). Anti-NLRP3 antibody (#A5652) was acquired from ABclonal Technology (Wuhan, China). Anti-α-SMA antibody (clone 1A4) and anti-LC3B antibody (L7543) were obtained from Sigma-Aldrich (Shanghai, China). Antibodies against p62 (#A19700), Beclin1 (#A11761), and ATG7 (#A0691) were obtained from ABconal (Wuhan, China). Horseradish peroxidase (HRP)-conjugated goat anti-mouse and goat anti-rabbit immunoglobulin G (IgG) were purchased from Proteintech (Wuhan, China).

### Histopathology

Liver tissue samples were collected and fixed (4% paraformaldehyde solution) at 4°C overnight, embedded with paraffin, and cut into 5 μm thick sections by sliding microtome (Leica, Shanghai, China). Oil Red O staining, HE staining, and Masson trichrome staining were performed to assess lipid accumulation, liver morphological characteristics, and liver fibrosis with standard procedure. The histological features were observed and imaged under the microscope (Leica, Shanghai, China), and the measurement of NAFLD activity score was based on the degree of steatosis, hepatocellular ballooning, and inflammation as previously described (Dyson et al., 2014).

### Immunohistochemistry Analysis

After being blocked with goat serum for antigen retrieval, the liver sections were incubated with primary antibodies against CD68 or *α*-SMA at 4°C overnight. The secondary antibody was applied to the sections after washing and incubated at room temperature for 1 h. The 3,3-diaminobenzidine (DAB) stained areas were observed and imaged under the microscope (Leica, Shanghai, China). The immunostained areas of liver sections were quantified with ImageJ software.

### Biochemical Analysis and Fatty Acids Assay

Alanine transaminase (ALT), aspartate transaminase (AST), lactate dehydrogenase (LDH), malondialdehyde (MDA), glutathione (GSH), glutathione peroxidase (GSH-px), and superoxide dismutase (SOD) levels were measured by commercial assay kits acquired from Nanjing Jiancheng Bioengineering Institute (Nanjing, China) according to the manufacturer’s instructions. Triglycerides (TG) levels were detected by the TG quantification kit (BioVision, Milpitas, United States). Hepatic fatty acids (palmitic, palmitoleic, and oleic acids) levels were analyzed as previously reported (Chen et al., 2014). The determination and quantification of PEA were performed as described by our previous study (Jin W. et al., 2020).

### ELISA Assays

The levels of TNF-*α*, MCP-1, RANTES, IL-1*β*, and IL-18 in each plasma sample were assessed by commercial ELISA kits (MultiSciences Biotech, Hangzhou, China) according to the manufacturer’s instructions.

### Western Blotting

Proteins from liver tissues were extracted and detected following a routine protocol, as previously reported (Hu et al., 2020). Briefly, proteins were loaded on SDS-PAGE gel for separation and transferred onto polyvinylidene fluoride membranes. These membranes were blocked in 5% fat-free milk at room temperature for 1 h and then incubated with anti-PPAR-*α* (1:1,000), anti-*α*-SMA (1:1,000), anti-LC3B (1:1,000), anti-p62 (1:1,000), anti-Beclin1 (1:1,000), anti-ATG7 (1:1,000), anti-NLRP3 (1:1,000), anti-caspase-1 (1:500), or anti-GAPDH (1:10,000) antibody overnight at 4°C. After washing with TBST three times, the membranes were incubated with a horseradish peroxidase-conjugated secondary antibody at room temperature for 1 h, following the visualization using an enhanced chemiluminescence agent. The quantitation of target proteins was performed by the ImageJ software and normalized to the GAPDH levels.

### Real-Time PCR

Total RNA was extracted from liver tissues using RNA simple Total RNA Kit (Tiangen, Beijing, China). The mRNA was used to synthesize cDNA by a FastQuant cDNA kit with gDNase (Tiangen, Beijing, China). The quantitation of mRNA was conducted with a SYBR Green kit (Tiangen, Beijing, China) according to the manufacturer’s instructions and normalized to GAPDH. The primer sequences are listed in Table 1.

**TABLE 1:** Primer sequence.

Genes	Primer sequence
ACC1	F: 5′-ATT​GGG​CAC​CCC​AGA​GCT​A-3′
	R: 5′-CCC​GCT​CCT​TCA​ACT​TGC​T-3′
PPAR-α	F: 5′-CCT​CAG​GGT​ACC​ACT​ACG​GAG​T-3′
	R: 5′-GCC​GAA​TAG​TTC​GCC​GAA-3′
CD36	F: 5′-ATG​GGC​TGT​GAT​CGG​AAC​TG-3′
	R: 5′-GTC​TTC​CCA​ATA​AGC​ATG​TCT​CC-3′
SOD1	F: 5′-AAC​CAG​TTG​TGT​TGT​CAG​GAC-3′
	R: 5′-CCA​CCA​TGT​TTC​TTA​GAG​TGA​GG-3′
SOD2	F: 5′-CAG​ACC​TGC​CTT​ACG​ACT​ATG​G-3′
	R: 5′-CTC​GGT​GGC​GTT​GAG​ATT​GTT-3′
TNF-α	F: 5′-CAG​GCG​GTG​CCT​ATG​TCT​C-3′
	R: 5′-CGA​TCA​CCC​CGA​AGT​TCA​GTA​G-3′
iNOS	F: 5′-AAT​CTT​GGA​GCG​AGT​TGT​GG-3′
	R: 5′-CAG​GAA​GTA​GGT​GAG​GGC​TTG-3′
RANTES	F: 5′-GCT​GCT​TTG​CCT​ACC​TCT​CC-3′
	R: 5′-TCG​AGT​GAC​AAA​CAC​GAC​TGC-3′
MCP-1	F: 5′-TTA​AAA​ACC​TGG​ATC​GGA​ACC​AA-3′
	R: 5′-GCA​TTA​GCT​TCA​GAT​TTA​CGG​GT-3′
MPO	F: 5′-GAC​ATG​CCC​ACC​GAA​TGA​CAA-3′
	R: 5′-CAG​GCA​ACC​AGC​GTA​CAA​AG-3′
TGF-β	F: 5′-TGA​CGT​CAC​TGG​AGT​TGT​ACG​G-3′
	R: 5′-GGT​TCA​TGT​CAT​GGA​TGG​TGC-3′
α-SMA	F: 5′-CAG​GCA​TGG​ATG​GCA​TCA​ATC​AC-3′
	R: 5′-ACT​CTA​GCT​GTG​AAG​TCA​GTG​TCG-3′
CTGF	F: 5′-GGG​CCT​CTT​CTG​CGA​TTT​C-3′
	R: 5′-ATC​CAG​GCA​AGT​GCA​TTG​GTA-3′
Col1a	F: 5′-ACG​GCT​GCA​CGA​GTC​ACA​C-3′
	R: 5′-GGC​AGG​CGG​GAG​GTC​TT-3′
Col3a	F: 5′-GTT​CTA​GAG​GAT​GGC​TGT​ACT​AAA​CAC​A-3′
	R: 5′-TTG​CCT​TGC​GTG​TTT​GAT​ATT​C-3′
GAPDH	F: 5′-CGG​TTC​CGA​TGC​CCT​GAG​GCT​CTT-3′
	R: 5′-CGT​CAC​ACT​TCA​TGA​TGG​AAT​TGA-3′

### Analysis of Statistics

All statistical analyses were performed using GraphPad Prism version 8.3.0 software. The experimental results are presented as the mean ± standard error of the mean (SEM). Statistical differences between multiple groups were analyzed using one-way analysis of variance (ANOVA) with Tukey’s multiple comparisons test. Statistical significance was set as * *p* < 0.05, ** *p* < 0.01, *** *p* < 0.001.

## Results

### PEA Attenuates MCD-Induced Steatohepatitis in Mice

To validate the effects of PEA in the development of NASH, PEA was administrated to MCD diet-induced NASH mice. The experimental scheme is shown in Figure 1A. MCD diet feeding caused a significant reduction of body weight, while PEA had no obvious impact on body weight compared with the MCD diet-fed mice during the experiment (Figure 1B). After sacrificing the mice, we first detected the role of PEA on MCD-induced NASH in mice through a histopathological evaluation, which is the gold standard for NASH assessment. HE staining showed that MCD diet-induced hepatic steatosis and ballooning compared to the mice who received a normal diet, while PEA treatment reduced both steatosis and ballooning (Figure 1C). Meanwhile, Oil Red O staining revealed that MCD diet feeding caused excessive lipid accumulation, while PEA significantly reduced the abundant lipid distribution in the liver (Figure 1D). Additionally, the high TG levels in the liver of MCD diet-treated mice were also obviously downregulated (Figure 1E). To further investigate the role of PEA on liver injury in MCD-treated mice, we determined the levels of ALT, AST, and LDH in plasma by a microplate-based method. MCD diet greatly induced the increase of plasma ALT, AST, and LDH levels, while PEA significantly decreased the levels of these biomarkers of liver function (Figures 1F–H). These results suggest that PEA significantly attenuated steatohepatitis in MCD diet-treated mice. In addition, we detected the levels of palmitic acid, palmitoleic acid, and oleic acid in the liver and found that palmitic acid levels were unchanged after being fed with a MCD diet, while hepatic palmitoleic acid and oleic acid levels were significantly decreased in mice fed MCD diet. Nevertheless, PEA treatment had no effect on all the above fatty acids compared with MCD-treated mice (Supplementary Figures S1A–C). Moreover, MCD diet feeding markedly elevated PEA levels, while PEA treatment enhanced this elevation compared to the Ctrl group (Supplementary Figure S1D). Meanwhile, we examined the expression of N-acylethanolamine-hydrolyzing acid amidase (NAAA) and fatty acid amide hydrolase (FAAH), both of which can hydrolyze endogenous PEA to palmitic acid and ethanolamine. The results showed that NAAA expression in the MCD group is higher than that in the Ctrl group and a reduced expression was observed in the PEA group, but these differences were not statistically significant (Supplementary Figure S1E). Notably, FAAH expression was obviously decreased with MCD feeding and unchanged after PEA treatment (Supplementary Figure S1F).

### PEA Regulates Gene Expression Involved in Lipid Metabolism

**FIGURE 2:**
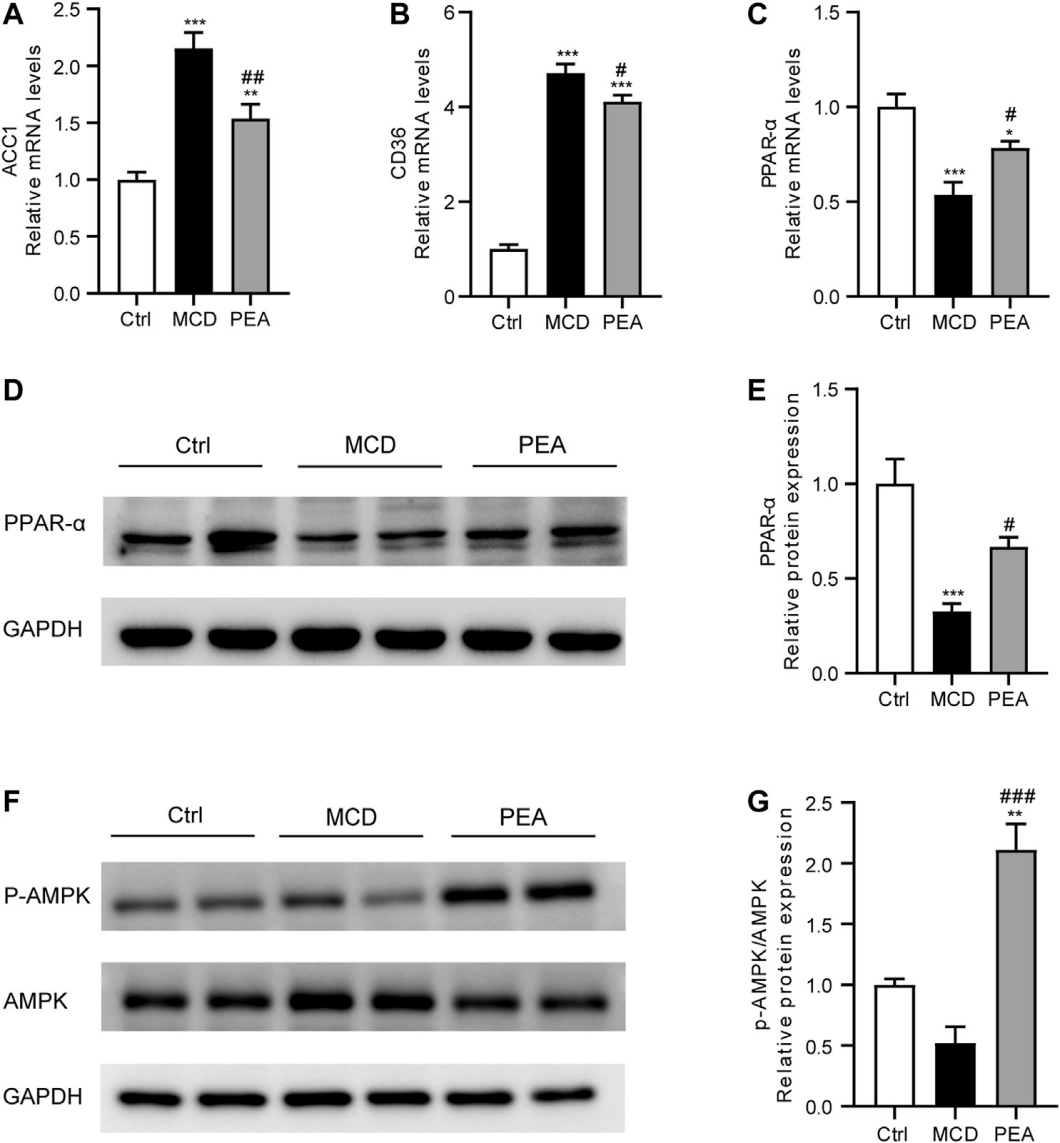
Effect of PEA on ACC1, CD36, PPAR-α and AMPK expression. **(A)** ACC1, **(B)** CD36, and **(C)** PPAR-α mRNA expression in the liver. **(D–E)** Hepatic PPAR-*α* protein levels were examined and quantified via western blotting. **(F–G)** Western blot determined p-AMPK and AMPK protein levels in the liver. Ctrl: the mice treated with standard diet; MCD: the mice treated with MCD diet; PEA: the mice treated with MCD diet and PEA. Values were expressed as the mean ± SEM, *n* = 6–8 for each group. *p < 0.05, **p < 0.01, ***p < 0.001 versus the Ctrl group; #*p* < 0.05, ##*p* < 0.01 versus the MCD group.

To explore the protective mechanism conferred by PEA on lipid accumulation in MCD-treated mice, we examined the gene expression related to lipid metabolism. As shown in Figures 2A, B, PEA treatment obviously reduced the mRNA expression of acetyl-CoA carboxylase 1 (ACC1) and CD36, which has a major effect on the biosynthesis and transporter of fatty acids, respectively. Moreover, PEA markedly upregulated the mRNA and protein expression levels of PPAR-*α*, which could promote fatty acid oxidation (Figures 2C–E). These results indicated that PEA could reduce lipid accumulation by decreasing ACC1 and CD36 and promoting the expression of PPAR-*α*. As the AMPK signaling pathway is the key metabolic pathway involved in regulating the above lipid metabolism associated genes, we further detected the protein levels of p-AMPK and AMPK, and the results suggested that the PEA could activate the AMPK signaling pathway in MCD-fed mice (Figures 2F, G).

### PEA Alleviates MCD-Induced Oxidative Stress

**FIGURE 3:**
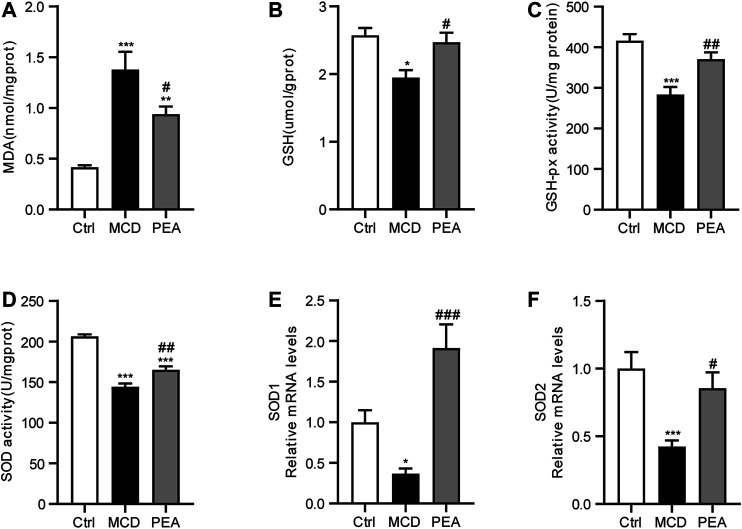
MCD-induced hepatic oxidative stress was alleviated in mice treated with PEA. **(A)** Levels of hepatic MDA, **(B)** levels of hepatic GSH, **(C)** activity of hepatic GSH-px, and **(D)** activity of hepatic SOD, **(E)** SOD1, and **(F)** SOD2 mRNA expression in liver. Ctrl: the mice treated with standard diet; MCD: the mice treated with MCD diet; PEA: the mice treated with MCD diet and PEA. Values were expressed as the mean ± SEM, *n* = 6–8 for each group. * *p* < 0.05, **p < 0.01, ***p < 0.001 versus the Ctrl group; #*p* < 0.05, ##*p* < 0.01, ###*p* < 0.001 versus the MCD group.

Oxidative stress is a common characteristic relative to the development of NASH (Chen et al., 2020). To assess the anti-oxidative activity of PEA, we detected the hepatic MDA levels, which is an important biomarker of oxidative stress (Zelber-Sagi et al., 2020). The data showed that the MCD diet obviously elevated the hepatic MDA levels, while PEA treatment significantly reduced MDA levels in the liver (Figure 3A). GSH is a powerful antioxidant in the liver, which plays a key role in radical scavenging (Delli Bovi et al., 2021). The results showed that MCD diet induced decreasing in GSH levels in the liver, which were markedly increased by the PEA treatment (Figure 3B). GSH-px and SOD are both enzymes with anti-oxidation activities to protect the liver from oxidative stress (Delli Bovi et al., 2021). We further found that the mice given the MCD diet showed reduced hepatic GSH-px and SOD activities, and PEA significantly reversed these changes (Figures 3C, D). Consistently, MCD diet feeding resulted in decreased mRNA levels of SOD1 and SOD2, and PEA treatment obviously increased SOD1 and SOD2 expression (Figures 3E, F). The above data showed that PEA treatment significantly alleviated oxidative stress in the liver.

### PEA Suppresses MCD-Induced Hepatic Inflammation

**FIGURE 4:**
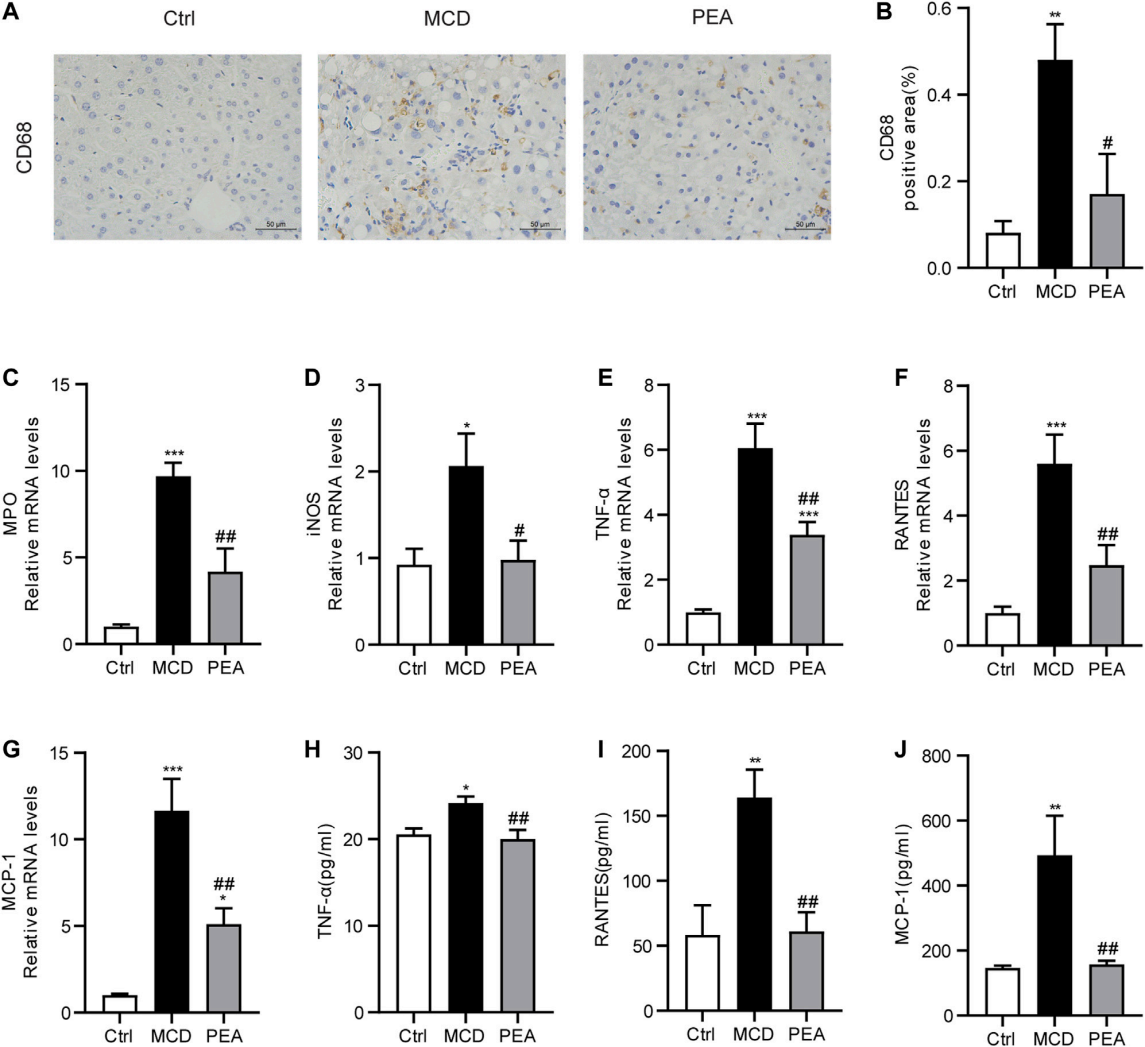
PEA ameliorated hepatic inflammation in mice fed by MCD diet. **(A,B)** Macrophage distribution in the liver was identified by staining of CD68 (× 400). The mRNA expression levels of **(C)** MPO, **(D)** iNOS, **(E)** TNF-α, **(F)** RANTES, and **(G)** MCP-1 in the liver measured *via* qRT-PCR. **(H–J)** The protein levels of TNF-α, RANTES, and MCP-1 in plasma detected by ELISA assay. Ctrl: the mice treated with standard diet; MCD: the mice treated with MCD diet; PEA: the mice treated with MCD diet and PEA. Values were expressed as the mean ± SEM, *n* = 6–8 for each group. *p < 0.05, **p < 0.01, ***p < 0.001 versus the Ctrl group; #*p* < 0.05, ##*p* < 0.01 versus the MCD group.

Inflammatory response is a key mechanism in the progress of NASH (Schuster et al., 2018). In order to verify the anti-inflammatory effects of PEA in MCD-induced NASH mice, we first detected the expression of CD68, a marker of Kupffer cells, via IHC staining. We found CD68 expression was greatly increased in liver tissues from MCD diet-treated mice, while PEA treatment significantly reduced this increase (Figure 4A,B). Next, we indicated that PEA dramatically attenuated MCD diet-induced production of inflammatory mediators, including MPO, iNOS, TNF-*α*, RANTES, and MCP-1 (Figures 4C–G). Moreover, the elevated expression of TNF-*α*, RANTES, and MCP-1 induced by MCD diet in plasma was also significantly decreased by PEA treatment (Figures 4H–J). These results suggested that PEA administration obviously inhibited hepatic inflammation in MCD diet-induced NASH mice.

### PEA Inhibits MCD-Induced NLRP3 Inflammasome Activation

**FIGURE 5:**
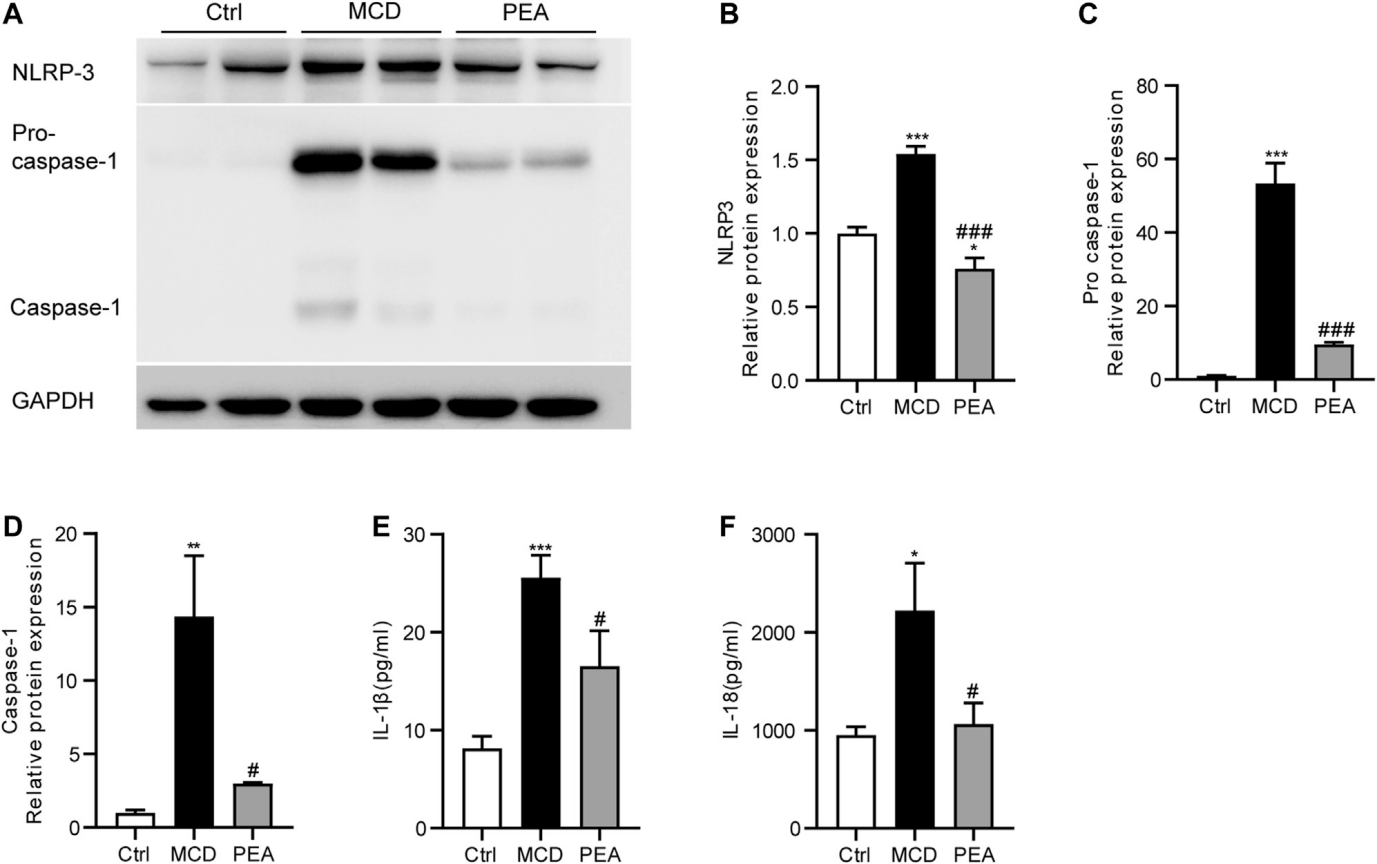
PEA attenuated MCD-induced NLRP3 inflammasome activation. **(A–D)** Key protein levels of the NLRP3 inflammasome pathway were analyzed *via* western blotting. **(E)** IL-1β and **(F)** IL-18 levels in plasma were measured using the ELISA method. Ctrl: the mice treated with standard diet; MCD: the mice treated with MCD diet; PEA: the mice treated with MCD diet and PEA. Values were expressed as the mean ± SEM, *n* = 6–8 for each group. *p < 0.05, **p < 0.01, ***p < 0.001 versus the Ctrl group; #*p* < 0.05, ###*p* < 0.001 versus the MCD group.

Several recent reports have demonstrated the crucial effects of NLRP3 inflammasome in the progression of NASH (Huang et al., 2021). To confirm the impact of PEA on the activation of the NLRP3 inflammasome, we examined the expression pattern of NLRP3, pro-caspase-1, and activated caspase-1 in the liver. As a result, MCD-induced elevated protein levels of NLRP3, pro-caspase-1, and activated caspase-1 were significantly inhibited by PEA treatment (Figures 5A–D). To further determine the potential role of PEA on the NLRP3 inflammasome pathway in MCD-treated NASH mice, we tested plasma IL-1*β* and IL-18 levels through ELISA assays. We found that plasma IL-1*β* and IL-18 levels in mice fed with MCD diet increased compared to those in mice fed with standard diet and were markedly reduced after PEA treatment (Figures 5E, F). The above results indicated that PEA could substantially block the NLRP3 signaling pathway in NASH mice fed with an MCD diet.

### PEA Restored Autophagy Signaling in MCD-Induced NASH Mice

**FIGURE 6:**
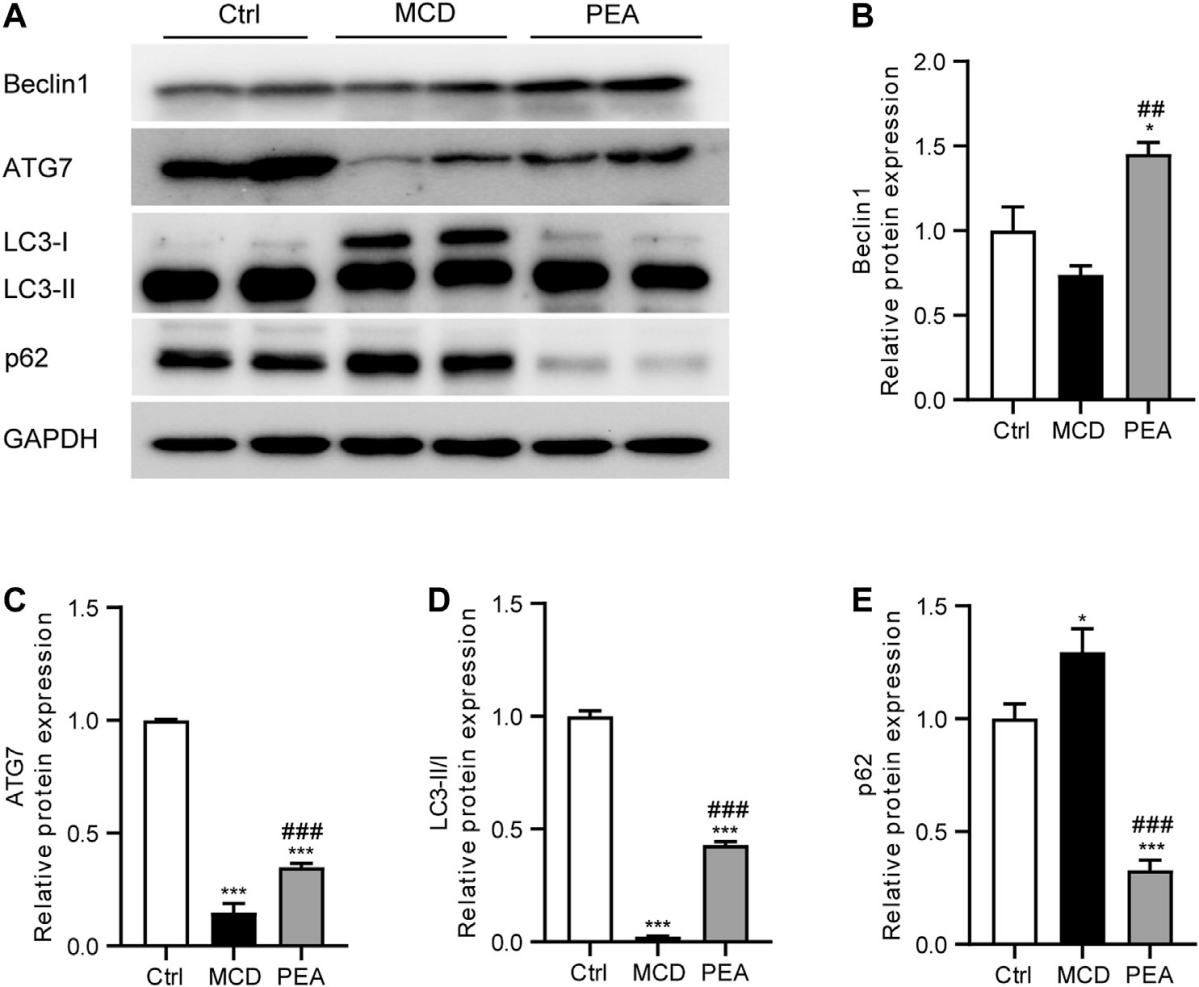
PEA enhanced autophagy during NASH. **(A–E)** Protein expression of hepatic Beclin1, ATG7, LC3II/I, and p62 levels. Ctrl: the mice treated with standard diet; MCD: the mice treated with MCD diet; PEA: the mice treated with MCD diet and PEA. Values were expressed as the mean ± SEM, *n* = 6–8 for each group. *p < 0.05, ***p < 0.001 versus the Ctrl group; ##*p* < 0.01, ###*p* < 0.001 versus the MCD group.

Numerous previous studies have indicated that autophagy plays an important part in the progress of NASH (Amir and Czaja, 2011; Allaire et al., 2019). To detect whether PEA regulates autophagy in mice who received MCD diet feeding, we analyzed the hepatic expression of Beclin-1, ATG7, LC3, and p62 via western blotting. We observed that PEA treatment greatly increased the protein levels of Beclin-1, ATG7, and LC3-II/LC3-I compared to the MCD group (Figures 6A–D). In addition, the low level of p62 protein in the MCD group was significantly upregulated after PEA treatment (Figure 6E). These data suggested that PEA reactivated autophagy signaling in MCD-treated mice.

### PEA Prevents MCD-Induced Liver Fibrosis

**FIGURE 7:**
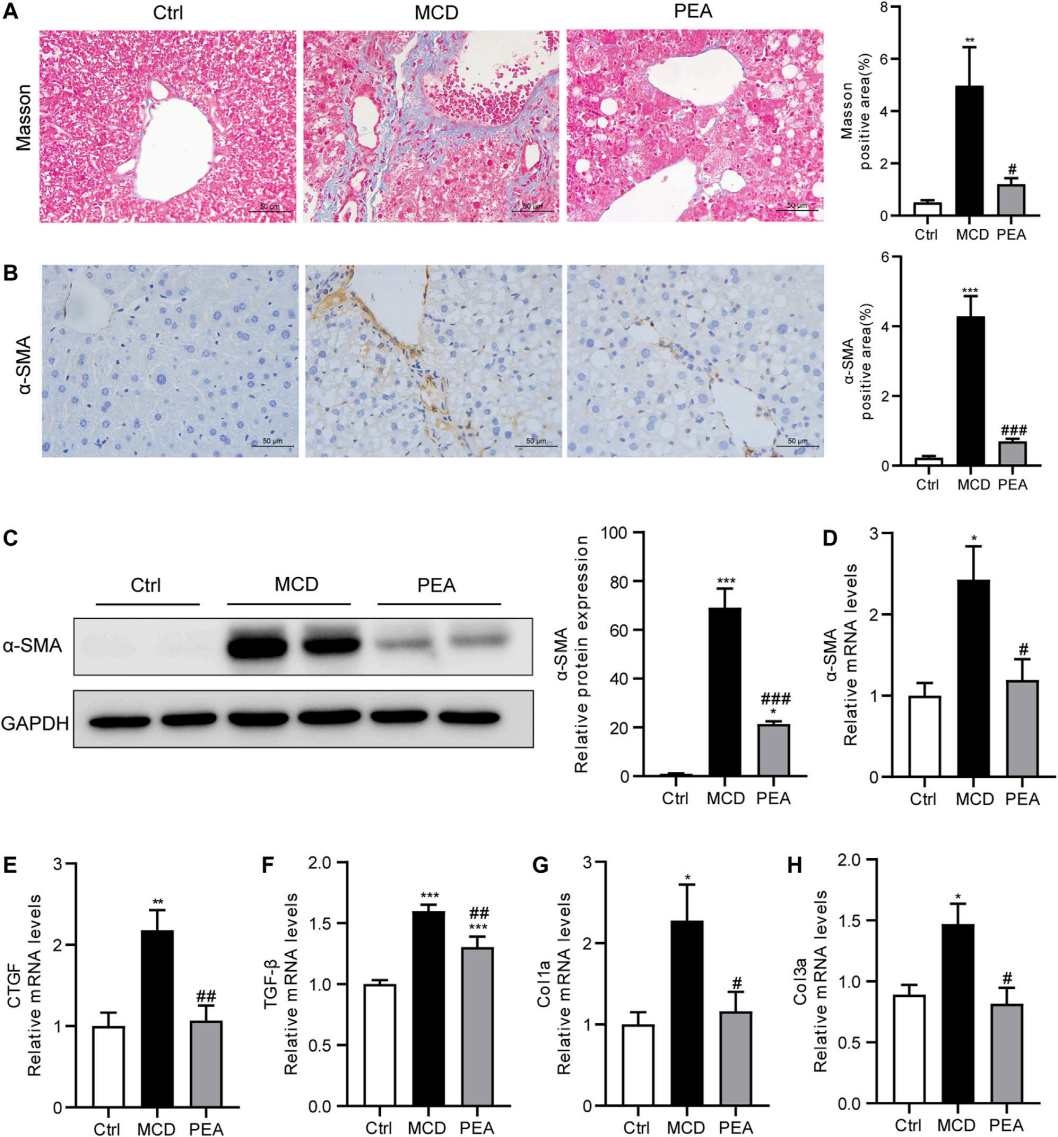
PEA attenuated hepatic fibrosis in mice who received MCD diet feeding. **(A)** Histological analysis of fibrosis in the liver via Masson’s trichrome staining (× 400). **(B)** Hepatic stellate cells activation was examined through staining of *α*-SMA in the liver (× 400). **(C)** Hepatic *α*-SMA protein levels determined by western blot. **(D)** *α* -SMA, **(E)** CTGF, **(F)** TGF-β, **(G)** Col1a, and **(H)** Col3a mRNA expression in liver. Ctrl: the mice treated with standard diet; MCD: the mice treated with MCD diet; PEA: the mice treated with MCD diet and PEA. Values were expressed as the mean ± SEM, *n* = 6–8 for each group. *p < 0.05, **p < 0.01, ***p < 0.001 versus the Ctrl group; #*p* < 0.05, ##*p* < 0.01, ###*p* < 0.001 versus the MCD group.

In mice fed the MCD diet, ongoing chronic liver injury ultimately results in liver fibrosis (Chen et al., 2015). To determine whether PEA affects MCD-induced nutritional fibrosis in mice, we measured the hepatic collagen deposition via Masson’s trichrome staining and found that the mice who received MCD diet feeding had much more collagen deposition than that of the control mice. However, the Masson staining area was obviously reduced in the PEA-treated mice (Figure 7A). *α*-Smooth muscle actin (*α*-SMA) is an important biomarker of hepatic stellate cells activation, which drives the progression of liver fibrosis. The protein levels of *α*-SMA were quantified by IHC staining and western blotting; the results revealed a marked increase of *α*-SMA expression in mice fed MCD diet compared to the mice given a normal diet, while PEA treatment obviously decreased the protein levels of *α*-SMA in comparison with the MCD group (Figures 7B, C). Moreover, PEA treatment also greatly decreased the MCD-induced mRNA expression of *α*-SMA (Figure 7D). Furthermore, we detected the mRNA levels of fibrotic genes, including CTGF, TGF-*β*, Col1a, and Col3a. The results demonstrated that the genes involved in liver fibrogenesis were significantly elevated in the MCD group compared with that in the control group, while PEA treatment obviously reversed these increases (Figures 7E–H). These results revealed the anti-fibrotic effects of PEA in MCD-induced NASH mice.

## Discussion

PEA is a bioactive molecule that has shown protective effects on various diseases, including neuropathy, colitis, and skin wounds (Petrosino and Di Marzo, 2017). However, little is known about its action in treating liver diseases, especially the protective role on NASH. The MCD diet feeding model used in our present study is one of the most well-recognized NASH models as it features the most important characteristics, including steatosis, inflammation, and fibrosis, which is similar to the pathogenetic course of human NASH (Machado et al., 2015). Here, a protective role of PEA in the progress of NASH was presented in this MCD-induced model for the first time. As we expected, we revealed that PEA effectively prevented hepatic lipid accumulation, hepatocellular ballooning, inflammation, and liver fibrosis. The mechanisms under the observations are mainly related to the anti-inflammatory effects of PEA, especially the inhibiting effect of PEA on the NLRP3 inflammasome pathway. Additionally, PEA reactivates autophagy signaling and plays an important role against oxidative stress.

Lipid metabolism dysfunction caused hepatic steatosis, which is a general pathological process in the initiation of NASH (Ipsen et al., 2018). Here, the protective role of PEA against hepatic steatosis is mainly implicated in the regulation of lipid metabolism genes. ACC1 is a cytosolic enzyme that converts acetyl-CoA into malonyl-CoA, which is catalyzed to form fatty acid and then causes the accumulation of triglycerides (TGs). Previous studies have revealed that ACC1 was highly expressed in NAFLD/NASH, while pharmacological inhibition of ACC1 offers a therapeutic approach for hepatic steatosis by inhibiting lipogenesis (Goedeke et al., 2018; Ross et al., 2020). CD36 is one of the best characterized mediators that promote the transport of fatty acids into cells. Abundant studies have demonstrated that the expression of hepatic CD36 is increased during the development of NAFLD with the capability of promoting hepatic fatty acids uptake, thus resulting in the enhancement of hepatic steatosis (Steneberg et al., 2015; Zhou et al., 2020). Consistent with previous reports, our study has shown increased mRNA expression of hepatic ACC1 and CD36 in MCD-induced NASH mice. However, PEA administration could obviously attenuate the expression of both ACC1 and CD36, suggesting that the anti-steatosis effect of PEA is partly dependent on the inhibition of ACC1-mediated lipogenesis and CD36-mediated fatty acids uptake. The nuclear receptor PPAR-*α* is the most important gene regulating fatty acid oxidation in hepatocytes (Montagner et al., 2016). PPAR-*α* ^−/−^ mice who received a MCD diet feeding showed more severe steatohepatitis than that in wild-type mice (Ip et al., 2003). Correspondingly, activation of PPAR-*α* could reduce hepatic lipid accumulation by promoting mitochondrial *β*-oxidation (Li et al., 2015; Pawlak et al., 2015). Our study has demonstrated that the decreased mRNA and protein expression of hepatic PPAR-*α* in MCD-induced NASH mice could be significantly upregulated after PEA treatment, suggesting that PEA may promote fatty acid *β*-oxidation through the PPAR-*α* signaling pathway. AMPK is a key regulator that controls energy homeostasis. Previous studies have indicated that AMPK activation could prevent the progress of NASH (Hu et al., 2021; Lan et al., 2021). Our data have shown that PEA significantly enhanced AMPK phosphorylation, suggesting that AMPK may be the center regulator of lipid metabolism in the current study. The effect of PEA on hepatic lipid metabolism has been investigated in a previous study, which revealed the protective activity of PEA to reduce hepatic lipid accumulation in the high-fat diet (HFD)-fed mice (Annunziata et al., 2020). The mechanistic findings in the above study are consistent with our present results that the beneficial effect of PEA on lipid metabolism may be associated with the activation of AMPK and PPAR-α signaling pathway.

Oxidative stress, which can promote the injury of hepatocytes, has been proved to be a crucial pathological event for the progression of NASH (Wang et al., 2020). In this research, we found that MCD diet feeding significantly increased the levels of MDA in the liver, while PEA treatment obviously attenuated this elevation. In addition, a massive downregulation of SOD and GSH-px activities was observed in MCD-treated mice, and an elevation effect was shown after PEA treatment. Therefore, reduced oxidative stress is likely responsible for improving liver injury during MCD feeding after PEA treatment.

Chronic inflammation plays an important role in the progression of NASH, which is mainly mediated *via* the expression and secretion of pro-inflammatory factors from intrahepatic macrophage (Kazankov et al., 2019). Annunziata et al. have revealed the anti-inflammatory action of PEA in HFD-induced steatosis mice (Annunziata et al., 2020). To evaluate the anti-inflammatory effect of PEA in steatohepatitis, we determined the action of PEA on inflammatory responses in MCD-treated mice. In the MCD-induced NASH mice, we observed increased hepatic CD68 expression, a marker of activated macrophage, associated with elevated MPO, iNOS, TNF-*α*, RANTES, and MCP-1 expression in liver and enhanced TNF-*α*, RANTES, and MCP-1 production in plasma. However, PEA treatment greatly diminished these inflammatory responses, suggesting that PEA has a powerful anti-inflammatory effect in the progression of NASH. Consistent with our results, PEA treatment in HFD-fed mice also significantly suppressed the expression of the pro-inflammatory factors, especially TNF-*α* in a previous study (Annunziata et al., 2020). The activation of NLRP3 inflammasome is also a vital mediator of NASH progression, which is closely related to the pathological progress of NASH, while blocking this signaling pathway has been reported as an efficient therapeutic approach to alleviate steatosis, inflammation, and even fibrosis in NASH model induced by MCD diet feeding (Mridha et al., 2017; Mahzari et al., 2019). Our study has shown that MCD diet feeding upregulated the expression of hepatic NLRP3, pro-caspase-1, and caspase-1, while PEA markedly decreased the levels of the above NLRP3 inflammasome related proteins. In the NLRP3 inflammasome activation process, caspase-1 could facilitate cleavage of the precursors of IL-1*β* and IL-18 to the mature forms, promoting inflammatory response as pro-inflammatory mediators. In our research, the data have indicated that PEA markedly eliminated the MCD-induced secretion of IL-1*β* and IL-18, which indicated that PEA can inhibit NLRP3 inflammasome activation during NASH development.

Autophagy is a natural course involved in lipid homeostasis, which could eliminate excessive intracellular lipids (Madrigal-Matute and Cuervo, 2016). The impaired autophagy signaling pathway has been reported to be closely related to the development of NASH (Chen et al., 2016). The agent that could activate autophagy may be a potential pharmacological application to prevent NASH (Jin X. et al., 2020). In our present study, we confirmed that MCD diet feeding impaired hepatic autophagy in mice and found that PEA reactivates autophagy as evidenced by elevated Bechin1, ATG7, and LC3-II/I levels and reduced p62 level. Therefore, we supposed that autophagy might be a very important mediator for the protective effects of PEA on MCD-induced NASH.

As is well known, steatohepatitis can progress to liver fibrosis (Chen et al., 2015; Cai et al., 2020). Herein, we also revealed the protective effect of PEA in the alleviation of liver fibrosis in NASH mice fed with a MCD diet. PEA has been recently reported to attenuate strabismus surgery-induced fibroproliferation to prevent postoperative adhesion through the suppression of canonical and non-canonical TGF-β signaling (Li et al., 2021). The expression of *α*-SMA, which is the most important biomarker of liver fibrosis, mainly regulated by the TGF-*β* signaling pathway (Kisseleva and Brenner, 2021). As our present study has demonstrated that PEA significantly alleviated the expression of TGF-*β*1 and *α*-SMA in the liver of MCD-treated mice, we speculate that the anti-fibrosis effect of PEA may be mainly dependent on the regulation of TGF-*β* signaling.

As an endogenous bioactive lipid, PEA can be degraded by NAAA and FAAH (Piomelli et al., 2020; Rankin and Fowler, 2020). Thus, increasing intracellular PEA accumulation through pharmacological inhibition of NAAA or FAAH could also be an efficient therapy in the modulation of various diseases (Wortley et al., 2017; Yang et al., 2020). Our previous reports have indicated that NAAA is an excellent target for anti-inflammatory and analgesic therapy (Yang et al., 2015; Jin W. et al., 2020). In our present study, we also examined hepatic PEA levels and analyzed the expression of FAAH and NAAA in the liver. As a result, MCD diet feeding significantly increased the levels of PEA in the liver, while hepatic PEA levels in the PEA treatment group are much higher than those in the MCD group. Since NAAA expression in the MCD group is upregulated, which is not significant, the MCD diet-induced elevation of PEA levels may be due to the greatly reduced expression of FAAH in the liver. Although NAAA and FAAH are differently expressed in MCD-induced NASH mice, whether NAAA or FAAH participated in the progression of NASH through regulation of the metabolism of PEA is still unknown. Therefore, there is still a need to investigate the endogenous expression of NAAA and PEA in all kinds of cells in the liver, such as hepatocytes, Kupffer cells, and hepatic stellate cells and to explore whether NAAA or FAAH inhibition has a beneficial effect on NASH.

In conclusion, our current study demonstrated that PEA could prevent the pathogenesis of steatohepatitis, as shown by reduced hepatic steatosis, oxidative stress, and liver fibrosis, which might be partly mediated *via* its powerful anti-inflammatory effect and the restoration of autophagy. Therefore, this finding provided new insights into the beneficial effect of PEA as a pharmacological agent affecting the development of NASH.

## Data Availability

The original contributions presented in the study are included in the article/Supplementary Material; further inquiries can be directed to the corresponding author.
