# Supplementary material for: Micronized Palmitoylethanolamide Ameliorates Methionine- and Choline-Deficient Diet–Induced Nonalcoholic Steatohepatitis via Inhibiting Inflammation and Restoring Autophagy
Source: Front Pharmacol. 2021 Oct 12;12:744483. doi: 10.3389/fphar.2021.744483 (PMC8546106; doi:10.3389/fphar.2021.744483)
Supplement: Supplementary file 1 [file DataSheet1.docx]

**Supplementary Table 1.** Composition of the normal diet and MCD diet formulas

| **Ingredients (g)** | **ND** | **MCD** |
| --- | --- | --- |
| Amino acids mixture without methionine | - | 176 |
| Amino acids mixture with methionine | 184 | - |
| Carbohydrate (dextrin, sucrose, corn starch) | 632 | 642 |
| Fiber (cellulose) | 30 | 30 |
| Fat (corn oil) | 100 | 100 |
| Mineral and vitamin mixture | 52 | 52 |
| Choline chloride | 2 | 0 |
| Antioxidant (TBHQ) | 0.02 | 0.02 |
|  |  |  |
| **Fatty acid content (% of total fat)** |  |  |
| Saturated fatty acid | 14.6% | 14.6% |
| Monounsaturated fatty acid | 28.4% | 28.4% |
| Polyunsaturated fatty acid | 57% | 57% |
|  |  |  |

**Supplementary Figure 1.**


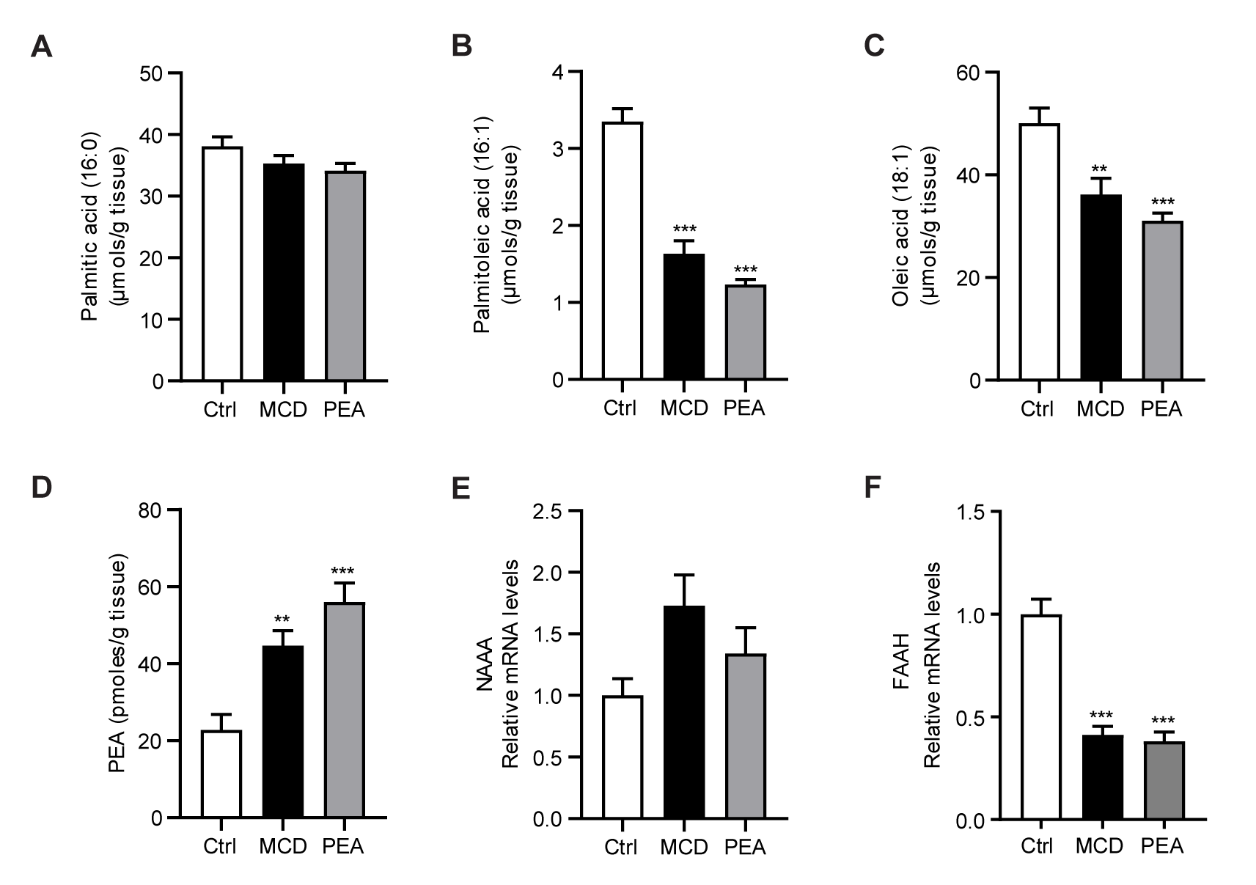


Supplementary Figure 1. (A) Levels of hepatic Palmitic acid (16:0). (B) Levels of hepatic Palmitoleic acid (16:1). (C) Levels of hepatic Oleic acid (18:1). (D) Levels of hepatic PEA. (E) NAAA and (F) FAAH mRNA expression in liver. Ctrl: The mice treated with standard diet; MCD: The mice treated with MCD diet; PEA: The mice treated with MCD diet and PEA. Values were expressed as the mean ± SEM, n = 6-8 for each group. ** P < 0.01, *** P < 0.001, versus the Ctrl group.
